# A poxviral-based cancer vaccine targeting the transcription factor Twist inhibits primary tumor growth and metastases in a model of metastatic breast cancer and improves survival in a spontaneous prostate cancer model

**DOI:** 10.18632/oncotarget.4442

**Published:** 2015-07-06

**Authors:** Anna R. Kwilas, Andressa Ardiani, Ulrike Dirmeier, Cornelia Wottawah, Jeffery Schlom, James W. Hodge

**Affiliations:** ^1^ Laboratory of Tumor Immunology and Biology, Center for Cancer Research, National Cancer Institute, National Institutes of Health, Bethesda, MD, USA; ^2^ Bavarian Nordic, Martinsried, Germany and Mountain View, CA, USA

**Keywords:** twist, vaccine, metastasis, TRICOM, immunotherapy

## Abstract

Several transcription factors play a role in the alteration of gene expression that occurs during cancer metastasis. Twist expression has been shown to be associated with the hallmarks of the metastatic process, as well as poor prognosis and drug resistance in many tumor types. However, primarily due to their location within the cell and the lack of a hydrophobic groove required for drug attachment, transcription factors such as Twist are difficult to target with conventional therapies. An alternative therapeutic strategy is a vaccine comprised of a Modified vaccinia Ankara (MVA), incorporating the Twist transgene and a TRIad of COstimulatory Molecules (B7-1, ICAM-1, LFA-3; TRICOM). Here we characterize an MVA-TWIST/TRICOM vaccine that induced both CD4+ and CD8+ Twist-specific T-cell responses *in vivo*. In addition, administration of this vaccine reduced both the primary tumor growth and metastasis in the 4T1 model of metastatic breast cancer. In the TRAMP transgenic model of spontaneous prostate cancer, MVA-TWIST/TRICOM alone significantly improved survival, and when combined with the androgen receptor antagonist enzalutamide, the vaccine further improved survival. These studies thus provide a rationale for the use of active immunotherapy targeting transcription factors involved in the metastatic process and for the combination of cancer vaccines with androgen deprivation.

## INTRODUCTION

The metastatic process is governed by multiple alterations in tumor cell phenotype and gene expression. Several transcription factors, including Twist, Snail, Slug and brachyury, have been found to play a role in the alteration of gene expression that occurs during metastasis [1–4]. Twist and brachyury, in particular, have been implicated in epithelial-mesenchymal transition (EMT), angiogenesis, inhibition of apoptosis and chromosomal instability, all of which are involved in the metastatic process [5–9]. In addition, studies have linked expression of Twist and brachyury to poor prognosis in multiple tumor types and to the generation of drug resistance [10–17]. Similarly, brachyury expression has been associated with EMT as well as poor prognosis in lung, colon and breast cancer, suggesting a need for targeted therapeutics [18–20]. However, the cellular location of transcription factors and their lack of a hydrophobic groove for drug binding make them difficult to target with conventional therapies. In addition, brachyury is neither expressed by normal murine tissues nor most murine tumor cell lines, making it difficult to evaluate the efficacy of vaccines targeting this transcription factor in preclinical murine tumor models [21]. Due to the similar expression profiles of brachyury and Twist, in their given species, and similar roles in metastatic progression, however, vaccines targeting Twist can serve as models for the use of vaccines targeting either Twist or brachyury in both monotherapy and combination therapy settings.

Modified vaccinia Ankara (MVA), a highly attenuated vaccinia virus with an extensive safety profile, is used as an alternative vaccine against smallpox. In Europe, MVA is approved for administration to all individuals, including those with compromised immune systems. Previously, we have demonstrated the ability of MVA to express the tumor-associated antigen carcinoembryonic antigen (CEA) as well as a TRIad of COstimulatory Molecules (TRICOM, consisting of B7-1, ICAM-1 and LFA-3). This cancer vaccine (MVA-CEA/TRICOM) induced significant antitumor immune responses [22]. MVA-based cancer vaccines have been evaluated clinically in lung, colon and renal cancer and are currently under investigation in breast and prostate cancer [23–27]. The TRICOM vaccine platform is currently being examined clinically in multiple cancer settings, including prostate, breast, colorectal, bladder and pancreatic carcinomas among other tumor types [28–31]. We sought to determine the ability of an MVA-TRICOM-based platform to express Twist and stimulate Twist-specific antitumor immune responses in two spontaneously metastasizing murine tumor models where Twist is a “self” tumor-associated antigen. Previous studies have shown low level Twist expression in normal murine tissues including lung, heart, muscle and spleen; however, Twist is overexpressed by most murine tumor cell lines including 4T1 and TRAMP cell lines [21]. Despite this expression pattern, no autoimmunity was observed when a yeast-based Twist vaccine was administered [32].

Here, we demonstrate the ability of MVA-TWIST/TRICOM to induce both CD4^+^ and CD8^+^ Twist-specific immune responses. The vaccine significantly reduced primary tumor growth and metastatic spread in the orthotopic 4T1 metastatic breast cancer model; in a setting of more advanced disease, MVA-TWIST/TRICOM also significantly reduced the degree of metastasis. In the TRAMP (transgenic adenocarcinoma of the mouse prostate) model of prostate cancer, MVA-TWIST/TRICOM treatment significantly increased overall survival in TRAMP transgenic (TRAMP-Tg) mice. This effect was amplified in this model when MVA-TWIST/TRICOM was combined with enzalutamide, an FDA-approved second-generation androgen receptor (AR) antagonist. These data support the ability to immunologically target tumor-associated transcription factors as well as the use of therapeutic cancer vaccines in combination with androgen deprivation therapy (ADT).

## RESULTS

### Transgenes are efficiently expressed in cells infected with MVA-TWIST/TRICOM

**Figure 1:**
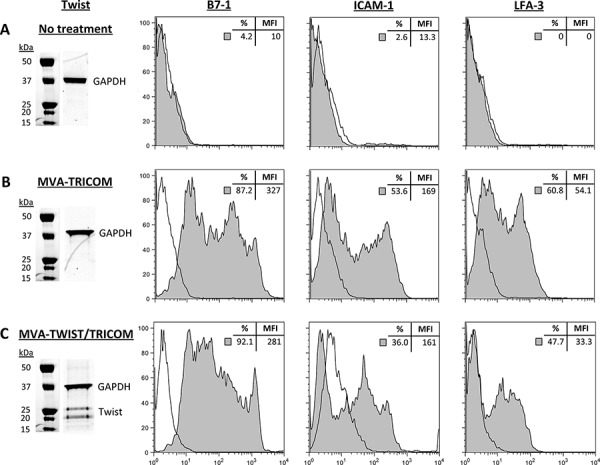
MVA-TWIST/TRICOM expresses Twist and a TRiad of COstimulatory Molecules (TRICOM) Western blot and flow cytometric analysis of MC38 cells treated with **A.** no treatment, **B.** MVA-TRICOM or **C.** MVA-TWIST/TRICOM for 24 hours. Antibodies against Twist and GAPDH were used for western blots. Antibodies against murine B7-1, ICAM-1, LFA-3 and isotype controls were used for flow cytometry. **Inserts:** percent of cells positive for the indicated marker and its mean fluorescent intensity (MFI). Data are representative of two independent experiments.

The efficiency of MVA-TWIST/TRICOM transgene expression was determined by western blot and flow cytometric analysis of infected MC38 murine tumor cells. MC38 cells were used to test the expression of these transgenes due to their lack of expression of Twist as well as the components of TRICOM (B7-1, ICAM-1, LFA-3). MC38 cells were infected with MVA-TWIST/TRICOM or MVA-TRICOM, or left untreated. Untreated MC38 cells showed no Twist expression by western blot as indicated by the absence of a doublet band at ∼26 and ∼21 kDa. Flow cytometric analysis of untreated MC38 cells indicated that the cells also lacked expression of murine B7-1, ICAM-1, and LFA-3 (Figure 1A). MC38 cells infected with MVA-TRICOM maintained a lack of Twist expression, again indicated by the lack of bands at ∼26 and ∼21 kDa in the western blot. However, these cells did display B7-1, ICAM-1 and LFA-3 expression as shown by histogram (Figure 1B). As expected, MC38 cells infected with MVA-TWIST/TRICOM displayed Twist expression, as shown by the presence of bands at ∼26 and ∼21 kDa in the western blot, as well as cell surface expression of B7-1, ICAM-1 and LFA-3 as determined by flow cytometry (Figure 1C). GAPDH was used as a loading control for the western blots.

### MVA-TWIST/TRICOM elicits Twist-specific T-cell responses in non-tumor bearing mice

**Figure 2:**
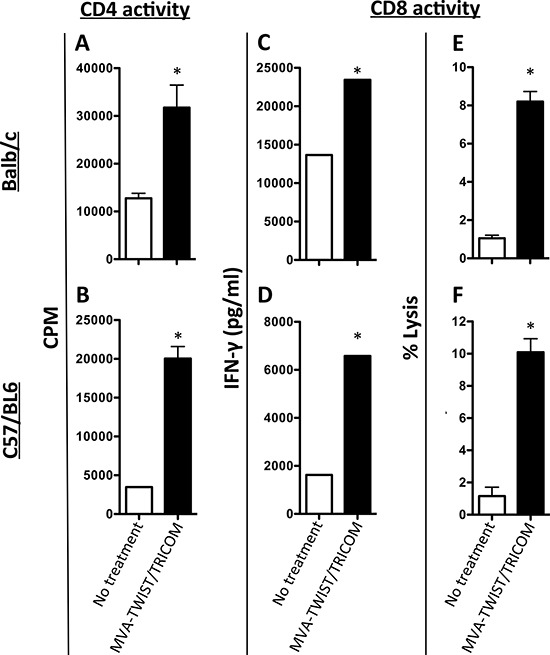
Administration of MVA-TWIST/TRICOM induces Twist-specific T-cell responses **A.** BALB/c mice and **B.** C57BL/6 mice were treated with three weekly doses of MVA-TWIST/TRICOM (black bars) or left untreated (open bars). The level of CD4^+^ T-cell proliferation was determined by [^3^H]-thymidine incorporation in the presence of irradiated antigen-presenting cells and Twist peptide. **C.** BALB/c mice and **D.** C57BL/6 mice were treated with three weekly doses of MVA-TWIST/TRICOM (black bars) or left untreated (open bars). The level of CD8^+^ T-cell activity was determined by the amount of secreted IFN-γ following 7 days of *in vitro* Twist peptide stimulation. The lytic ability of the CD8^+^ T cells from **E.** BALB/c mice and **F.** C57BL/6 mice treated with MVA-TWIST/TRICOM (black bars) or left untreated (open bars) was evaluated against peptide-pulsed p815 target cells. Error bars indicate mean ± S.E.M. from triplicate measurements. Statistical analyses for CD4^+^ T-cell proliferation were done by Student’s *t*-test, * = *P* < 0.05. For IFN-γ secretion, significance was determined by the Kolmogorov-Smirnov test. Data are representative of three independent experiments.

The ability of MVA-TWIST/TRICOM to induce Twist-specific T-cell responses was evaluated in both BALB/c and C57BL/6 mice where Twist is a “self” antigen. To determine if MVA-TWIST/TRICOM could induce Twist-specific CD4^+^ T-cell responses, BALB/c and C57BL/6 mice were vaccinated at weekly intervals three times with MVA-TWIST/TRICOM or left untreated. Three weeks following the last vaccination, mice were euthanized; their splenic CD4^+^ cells were purified and plated with antigen-presenting cells (APCs) from naïve mice in the presence of Twist peptide. CD4^+^ T cells from BALB/c (Figure 2A) as well as C57BL/6 (Figure 2B) mice vaccinated with MVA-TWIST/TRICOM proliferated to a significantly greater extent in the presence of Twist peptide (*P* = 0.0151 and 0.0038, respectively) than those from untreated mice. To assess the ability of MVA-TWIST/TRICOM to induce Twist-specific CD8^+^ T cell responses, BALB/c or C57BL/6 mice were vaccinated at weekly intervals three times with MVA-TWIST/TRICOM or MVA-TRICOM, or left untreated. Three weeks following the last vaccination, mice were euthanized and their splenocytes were harvested and stimulated with Twist peptide. IFN-γ production by CD8^+^ T cells was measured following restimulation. In both BALB/c and C57BL/6 mice there was a significant increase in IFN-γ secretion from CD8^+^ T cells from MVA-TWIST/TRICOM vaccinated vs. control mice (Figure 2C and 2D, respectively). In MVA-TWIST/TRICOM-treated BALB/c mice, IFN-γ secretion increased 1.7-fold. In treated C57BL/6 mice, secretion increased 4-fold compared to that of untreated mice. Administration of MVA-TRICOM alone also induced a significant increase in IFN-γ production in response to Twist peptide from CD8^+^ T cells from both BALB/c and C57BL/6 mice; however, this increase was significantly lower than the levels seen with MVA-TWIST/TRICOM vaccination (data not shown). In addition, following restimulation, CD8^+^ T cells from MVA-TWIST/TRICOM-vaccinated BALB/c mice (Figure 2E, *P* = 0.0002) and C57/BL6 mice (Figure 2F, *P* = 0.025) were able to lyse target cells pulsed with Twist peptide to a significantly greater degree than CD8^+^ T cells from untreated animals.

The effect of MVA-TWIST/TRICOM on the presence of Twist-specific, activated and memory immune cells in the spleens of BALB/c mice was examined further by flow cytometry. BALB/c mice were vaccinated twice at weekly intervals with MVA-TWIST/TRICOM or left untreated. Seventeen days following the last vaccination, mice were euthanized and their spleens were evaluated for immune cell composition. Analysis indicated that while the percentage of CD4^+^ T cells, CD8^+^ T cells and dendritic cells did not change, the percentage of effector memory CD8^+^ T cells, Twist-specific CD8^+^ T cells and activated dendritic cells did significantly increase after vaccination with MVA-TWIST/TRICOM (Table 1).

**Table 1:** Splenic immune cell subset changes in non-tumor bearing BALB/c mice after administration of MVA-TWIST/TRICOM

	CD4+ T cells	CD8+ T cells	Effector Memory CD8+ T cells	Twist Tetramer+ CD8+ T cells	Dendritic cells	Activated Dendritic cells
**No Treatment**	26.10 (1.017)	10.92 (0.2853)	0.7470 (0.0473)	2.43	1.390 (0.1243)	0.2592 (0.0762)
**MVA-TWIST/TRICOM**	26.78 (0.5093)	12.22 (0.2131)	1.56 (0.1597)*	3.83*	1.410 (0.07629)	0.5564 (0.0702)*

Flow cytometric analysis of splenic immune cell subsets in BALB/c mice (*n* = 5/group). Animals were administered two weekly vaccinations with MVA-TWIST/TRICOM or left untreated. Spleens were harvested 17 days after the last vaccination. Data represented as “mean (SEM)” percentage of viable splenocytes. CD4+ T cells (CD3+ CD4+), CD8+ T cells (CD3+ CD8+), Effector Memory cells (CD3+CD8+CD44+CD62L-), Dendritic cells (CD11c+MHCII+) and Activated Dendritic cells (CD11c+MHCII+CD40+).

* = *p* < 0.01 as determined by Student’s *t* test or the Kolmogorov-Smirnov test.

### MVA-TWIST/TRICOM elicits Twist-specific T-cell responses in 4T1-tumor bearing mice

It is largely accepted that the presence of cancer causes immune dysfunction in the host [33, 34]. The effect of MVA-TWIST/TRICOM on the composition of immune cells in the spleens of tumor bearing BALB/c mice was examined by flow cytometry. Four days following orthotopic implantation of 4T1 breast cancer cells, BALB/c mice were administered two weekly vaccinations with MVA-TWIST/TRICOM or left untreated. Seventeen days following the last vaccination, mice were euthanized and their splenocytes were harvested and evaluated for immune cell composition. The percentage of CD4^+^ T cells and CD8^+^ T cells as well as effector memory CD8^+^ T cells and central memory CD8^+^ T cells did significantly increase after vaccination with MVA-TWIST/TRICOM (Table 2). However, there was no alteration in the percentage of dendritic cells, activated dendritic cells, T regulatory cells, myeloid derived suppressor cells or natural killer cells (Table 2 and data not shown).

**Table 2:** Splenic immune cell subset changes in 4T1-tumor bearing BALB/c mice after administration of MVA-TWIST/TRICOM

	CD4+ T cells	CD8+ T cells	Effector Memory CD8+ T cells	Central Memory CD8+ T cells	Dendritic cells	Activated Dendritic cells
**No Treatment**	7.854 (1.396)	3.212 (0.4886)	0.5732 (0.116)	1.182 (0.1463)	1.090 (0.1974)	0.3352 (0.0745)
**MVA-TWIST/TRICOM**	14.10 (1.684)*	6.224 (0.9233)*	1.411 (0.2882)*	2.484 (0.4521)*	1.166 (0.1646)	0.3026 (0.0736)

Flow cytometric analysis of splenic immune cell subsets in 4T1-tumor bearing BALB/c mice (*n* = 5/group). Four days post-tumor implantation, mice were administered two weekly vaccinations with MVA-TWIST/TRICOM or left untreated. Spleens were harvested 17 days after the last vaccination. Data represented as “mean (SEM)” percentage of viable splenocytes. CD4+ T cells (CD3+ CD4+), CD8+ T cells (CD3+ CD8+), Effector Memory cells (CD3+CD8+CD44+CD62L-), Central Memory cells (CD3+CD8+CD44+CD62L+), Dendritic cells (CD11c+MHCII+) and Activated Dendritic cells (CD11c+MHCII+CD40+).

* = *p* < 0.01 as determined by Student’s *t* test.

**Figure 3:**
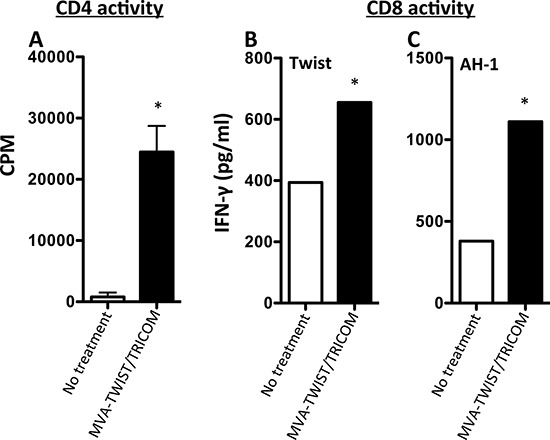
Treatment with MVA-TWIST/TRICOM induces Twist-specific T-cell responses in 4T1 tumor-bearing BALB/c mice BALB/c mice (*n* = 5/group) were implanted s.c. with 5 × 10^4^ 4T1 cells. Four days post-tumor implantation, mice received the first of two weekly MVA-TWIST/TRICOM (black bars) or were left untreated (open bars). **A.** The level of CD4^+^ T-cell proliferation was determined by [^3^H]-thymidine incorporation in the presence of irradiated antigen-presenting cells and Twist peptide. **B.** The level of CD8^+^ T-cell activity was determined by the amount of secreted IFN-γ following 7 days of *in vitro* Twist peptide stimulation. **C.** The level of CD8^+^ T-cell activity was determined by the amount of secreted IFN-γ following 7 days of *in vitro* AH-1 peptide stimulation. Error bars indicate mean ± S.E.M. from triplicate measurements. Statistical analyses for CD4^+^ T-cell proliferation were done by Student’s *t*-test, * = *P* < 0.05. For IFN-γ secretion, significance was determined by the Kolmogorov-Smirnov test.

The ability of MVA-TWIST/TRICOM to induce Twist-specific T-cell responses in the presence of tumor was also evaluated in the BALB/c 4T1 orthotopic breast cancer model. CD4^+^ cells were purified from harvested splenocytes and plated with naïve APCs in the presence of Twist peptide. CD4^+^ T cells from vaccinated BALB/c mice (Figure 3A) proliferated to a significantly greater extent in the presence of Twist peptide (*P* = 0.0054) than those from untreated mice. Non-purified splenocytes were stimulated with a Twist peptide or a non-vaccine encoded AH-1 peptide for 7 days after which IFN-γ production was measured. A significant increase in IFN-γ secretion was observed from CD8^+^ T cells from MVA-TWIST/TRICOM vaccinated vs. control mice in response to Twist peptide (Figure 3B). Increased IFN-γ secretion was also observed from CD8^+^ T cells from MVA-TWIST/TRICOM vaccinated mice in response to AH-1 peptide (Figure 3C). IFN-γ secretion increased 1.7-fold with Twist peptide stimulation and 2.9-fold with AH-1 peptide stimulation.

### MVA-TWIST/TRICOM vaccination improves T-cell infiltration into the tumor microenvironment

**Figure 4:**
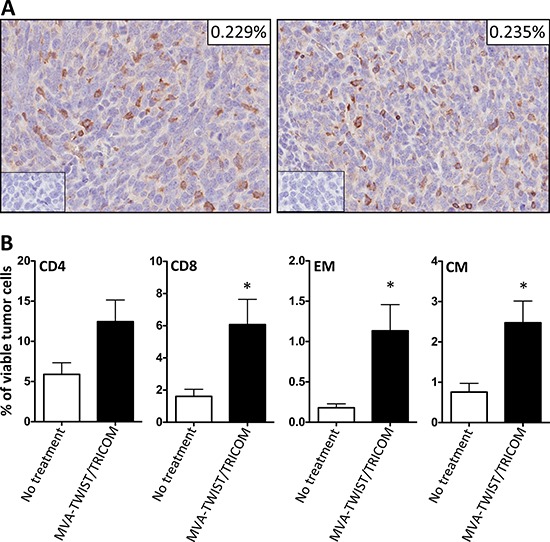
MVA-TWIST/TRICOM administration improves tumor infiltration of immune effector cells BALB/c mice (*n* = 5/group) were implanted s.c. with 5 × 10^4^ 4T1 cells. Four days post-tumor implantation, mice received the first of two weekly MVA-TWIST/TRICOM vaccinations or were left untreated. Seventeen days following the last vaccination, tumors were harvested and analyzed by immunohistochemistry and flow cytometry. **A.** CD3^+^ lymphocyte infiltration as determined by immunohistochemistry. **Inserts:** Isotype control staining and percentage of tumor composed of CD3^+^ cells. **B.** Percentage of CD4^+^ T cells (CD3+ CD4+), CD8^+^T cells (CD3+ CD8+), Central Memory (CM, CD3+ CD8+CD44+CD62L+) and Effector Memory (EM, CD3+ CD8+CD44+CD62L-) cells present in the tumor as determined by flow cytometric analysis. Error bars indicate mean ± S.E.M. from five measurements. Statistical analyses were done by Student’s *t*-test, * = *P* < 0.05.

Since MVA-TWIST/TRICOM altered the composition of splenic immune cell populations in 4T1 tumor bearing BALB/c mice, we sought to determine if this translated into improved infiltration of immune cells into the tumor microenvironment. Four days following implantation of 4T1 cells, BALB/c mice were administered two weekly vaccinations with MVA-TWIST/TRICOM or left untreated. Seventeen days following the last vaccination, mice were euthanized and their tumors were harvested and evaluated for immune cell infiltration by immunohistochemistry and flow cytometry. There was no difference in the tumoral infiltration of CD3^+^ lymphocytes (*P* = 0.8622) between MVA-TWIST/TRICOM and untreated animals as determined by immunohistochemistry and confirmed by flow cytometry (Figure 4A and data not shown). Tumors from mice treated with MVA-TWIST/TRICOM also did not show a significant increase in CD4^+^ T-cell infiltration compared to tumors from untreated mice (Figure 4B, *P* = 0.0567); however, they did show a significant increase in the number of infiltrating CD8^+^ T cells (Figure 4B, *P* = 0.0185). Tumors from mice treated with MVA-TWIST/TRICOM also displayed increased infiltration of effector memory (*P* = 0.0135) and central memory (*P* = 0.0144) CD8+ T cells compared to tumors from untreated mice (Figure 4B). MVA-TWIST/TRICOM administration did not alter the infiltration of T regulatory cells, myeloid derived suppressor cells, natural killer cells or dendritic cells as determined by flow cytometric analysis (data not shown). Taken together, these data indicate that MVA-TWIST/TRICOM induces a more immune-stimulatory environment, both in the periphery and at the tumor site.

### MVA-TWIST/TRICOM vaccination inhibited primary tumor growth and metastases

**Figure 5:**
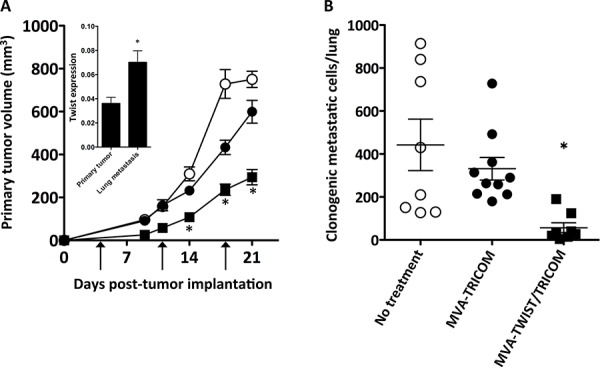
Treatment with MVA-TWIST/TRICOM inhibits primary tumor growth and spontaneous lung metastasis in the 4T1 metastatic breast cancer model BALB/c mice (*n* = 10/group) were implanted s.c. with 5 × 10^4^ 4T1 cells. Four days post-tumor implantation the first of three weekly vaccinations was given, as indicated by arrows. **A.** Primary tumor growth in mice treated with MVA-TWIST/TRICOM (closed squares) or MVA-TRICOM (closed circles) or left untreated (open circles). **Insert:** Twist expression in 4T1 primary tumor and lung metastases as determined by RT-PCR (*n* = 3). **B.** Enumeration of clonogenic metastatic cells in the lungs of mice treated with MVA-TWIST/TRICOM (closed squares) or MVA-TRICOM (closed circles) or left untreated (open circles) obtained 21 days post-tumor implantation. Tumor dimensions were measured weekly. Clonogenic metastatic cells/lung were determined by plating a single cell suspension of lung cells in the presence of 6-thioguanine for 10–14 days after which colonies were enumerated. Error bars indicate mean ± S.E.M. Statistical analyses were done by Student’s *t*-test, * = *P* < 0.005 vs. either no treatment or MVA-TRICOM alone. Data are representative of three independent experiments.

The ability of MVA-TWIST/TRICOM to inhibit tumor growth and metastasis was also evaluated in the BALB/c 4T1 orthotopic breast cancer model as Twist has been shown to play a role in metastatic spread in this model [1]. Twist expression was validated by RT-PCR in primary tumor tissue as well as lung metastases from untreated mice. Twist expression was significantly higher (*P* = 0.0083) in the lung metastases of 4T1 tumor-bearing mice compared to that of the primary tumors (Figure 5A, insert). This upregulation of Twist expression in the metastases suggests that MVA-TWIST/TRICOM could have a greater effect on the inhibition of metastasis than primary tumor growth in this model. 4T1 tumor-bearing mice were given three weekly doses of MVA-TWIST/TRICOM or MVA-TRICOM starting 4 days post-tumor implantation, when tumors become palpable, or left untreated. Twenty-one days after tumor implantation, mice were euthanized and their lungs were harvested for examination of metastasis. Mice receiving MVA-TWIST/TRICOM displayed significantly reduced primary tumor growth compared to mice receiving either no treatment or MVA-TRICOM (Figure 5A). More strikingly, mice receiving MVA-TWIST/TRICOM had significantly fewer clonogenic metastatic cells in their lungs compared to mice receiving either no treatment (*P* = 0.0036) or MVA-TRICOM (*P* = 0.0004) (Figure 5B). There was no toxicity associated with treatment.

### MVA-TWIST/TRICOM vaccination maintained anti-metastatic efficacy in more advanced disease

**Figure 6:**
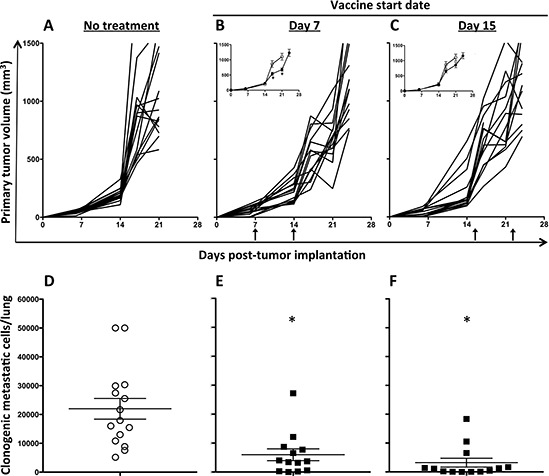
MVA-TWIST/TRICOM administration reduces the formation of lung metastases when treatment is initiated up to 15 days post-tumor implantation BALB/c mice (*n* = 15/group) were implanted s.c. with 5 × 10^4^ 4T1 cells. Graphs show primary tumor growth in mice treated with **A.** no treatment, **B.** MVA-TWIST/TRICOM beginning 7 days post-tumor implantation or **C.** MVA-TWIST/TRICOM beginning 15 days post-tumor implantation. Vaccinations are indicated by arrows. **Inserts:** Average tumor growth per group compared to group receiving no treatment. Enumeration of clonogenic metastatic cells, obtained when the group average tumor volume reached 1000mm^3^, in the lungs of mice treated with **D.** no treatment, **E.** MVA-TWIST/TRICOM beginning on day 7 post-tumor implantation or **F.** MVA-TWIST/TRICOM beginning on day 15 post-tumor implantation. Tumor dimensions were measured weekly. Clonogenic metastatic cells were determined as in previous experiment. Error bars indicate mean ± S.E.M. Statistical analyses were done by Student’s *t*-test, * = *P* < 0.01 vs. no treatment. Data are representative of two independent experiments.

Delaying treatment in the 4T1 model provides more time for primary as well as metastatic disease to progress, making it a more stringent model for evaluating treatments in the setting of advanced disease. To determine if postponing MVA-TWIST/TRICOM vaccination would reduce its antitumor efficacy, 4T1 tumor-bearing mice were given two weekly doses of MVA-TWIST/TRICOM starting 7 or 15 days post-tumor implantation or left untreated. Treatment groups in this study were euthanized and their lungs were harvested when the average tumor volume of the group breached 1000 mm^3^. Examining the mice for metastatic disease when primary tumor volumes were equivalent eliminated the possibility that reduced metastatic disease was due to smaller primary tumors. Figure 6 shows the growth of individual tumors in mice receiving no treatment (panel A) or MVA-TWIST/TRICOM beginning 7 (panel B) or 15 (panel C) days post-tumor implantation. Prior to receiving treatment on either day 7 or day 15 post-tumor implantation, tumor volumes were equivalent between the treated and untreated groups. Despite a setting of increased tumor burden when initiating MVA-TWIST/TRICOM 7 days post-implantation, primary tumor growth was still reduced (Figure 6B, insert). Delaying vaccination to day 15, however, eliminated this effect on the primary tumor (Figure 6C, insert). In addition to significantly reducing primary tumor growth when administered 7 days post-implantation, MVA-TWIST/TRICOM also significantly (*P* = 0.001) impacted metastatic disease (Figure 6E). When administered 15 days post-tumor implantation, even though MVA-TWIST/TRICOM did not significantly inhibit primary tumor growth, vaccination maintained a significant reduction in clonogenic metastatic cells (*P* = 0.0001) (Figure 6F). There was no significant difference in the reduction of primary or metastatic disease between mice treated with MVA-TWIST/TRICOM 7 days or 15 days post-tumor implantation.

### MVA-TWIST/TRICOM significantly increased the survival of TRAMP-Tg mice when administered alone or in combination with enzalutamide

**Figure 7:**
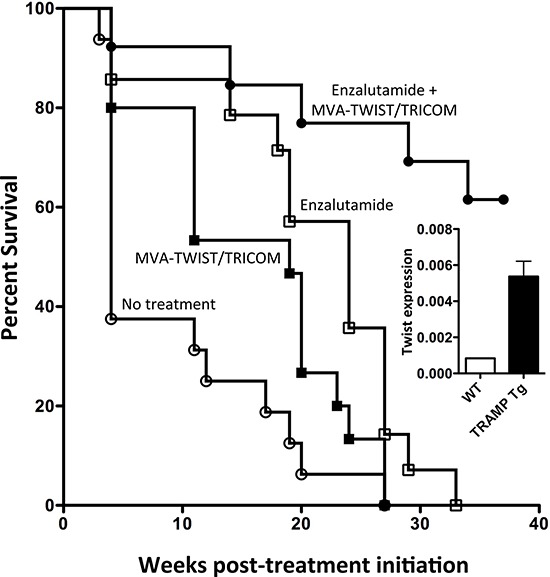
Combining MVA-TWIST/TRICOM with anti-androgen therapy significantly increases survival in Tramp-Tg mice Tramp-Tg mice were age-matched and randomized to receive one of the indicated treatment regimens. Indicated mice began receiving enzalutamide and received the first of three weekly vaccinations with MVA-TWIST/TRICOM simultaneously on day 0. **A.** Overall survival of Tramp-Tg mice receiving no treatment (open circles), MVA-TWIST/TRICOM (closed squares), enzalutamide (open squares) or the combination of MVA-TWIST/TRICOM and enzalutamide (closed circles). **Insert:** Twist expression in the prostates of wild-type C5/BL6 and Tramp-Tg mice as determined by RT-PCR (*n* = 12). Statistical analyses were done by Student’s *t*-test relative to indicated group.

The TRAMP model of spontaneous prostate cancer, wherein tumor development resembles disease progression in humans, from prostatic intraepithelial neoplasia (PIN) to metastatic castration-resistant prostate cancer (CRPC) [35–38], provided a second tumor model to evaluate the antitumor efficacy of MVA-TWIST/TRICOM. The expression of Twist in the prostates of TRAMP-Tg mice was validated by RT-PCR. Analysis indicated that Twist expression was significantly higher in the prostates of TRAMP-Tg mice than in prostates of wild-type C57BL/6 mice, suggesting a rationale for the use of this vaccine in this model (Figure 7A, insert). In this study, age-matched groups of TRAMP-Tg mice were treated with MVA-TWIST/TRICOM with or without enzalutamide, an FDA-approved androgen receptor (AR) antagonist and first-line therapy for men with metastatic CRPC [39]. Mice receiving MVA-TWIST/TRICOM alone displayed a significant increase in median survival compared to untreated controls (19 vs. 4 weeks, *P* = 0.048), as did mice receiving enzalutamide alone (24 vs. 4 weeks, *P* = 0.001, Figure 7). Mice receiving the combination of MVA-TWIST/TRICOM and enzalutamide also exhibited a significant increase in median survival compared to untreated animals (>36 vs. 4 weeks, *P* < 0.0001) as well as compared to mice receiving MVA-TWIST/TRICOM (>36 vs. 19 weeks, *P* < 0.0001) or enzalutamide (>36 vs. 24 weeks, *P* = 0.0004) alone (Figure 7). Median survival of the group receiving the combination of MVA-TWIST/TRICOM and enzalutamide was not met by 40 weeks post-treatment initiation, when animals in all other groups were deceased (Figure 7). There was no toxicity associated with treatment.

## DISCUSSION

A recent report described the ability of a recombinant yeast-based vaccine to induce an immune response against the self-antigen Twist [32]. Those studies indicated that yeast-Twist induced an antitumor immune response in the 4T1 model in the absence of autoimmunity, even though Twist expression was confirmed in normal tissue of BALB/c mice. The yeast and pox-TRICOM platforms have previously been shown to be quite different in terms of the types of T cells and cytokines induced [40–42]. Here we sought to confirm that metastasis-associated transcription factors are suitable targets for vaccine-mediated immunotherapy. We utilized two mouse strains, BALB/c and C57BL/6, and two tumor models, the 4T1 metastatic breast cancer model and TRAMP-Tg spontaneous prostate cancer model, to evaluate whether the MVA-TWIST/TRICOM vaccine platform could induce anti-Twist immune responses leading to antitumor efficacy.

We first sought to determine if MVA-TWIST/TRICOM could induce Twist-specific CD4^+^ and CD8^+^ T-cell responses. We observed that both BALB/c and C57BL/6 mice treated with MVA-TWIST/TRICOM exhibited significantly increased CD4^+^ T-cell proliferation in response to Twist peptide compared to that of untreated mice (Figure 2A and 2B). In addition, mice receiving MVA-TWIST/TRICOM demonstrated significantly increased CD8^+^ T cell-mediated IFN-γ production in response to Twist peptide (Figure 2C and 2D). As indicated by the difference in scale, both vaccinated and unvaccinated BALB/c mice exhibited significantly greater Twist-specific T-cell responses compared to those of C57BL/6 mice (Figure 2A and 2C). This observation could be due to slight differences in the peptides used to stimulate the T cells of the different mouse strains or to the possible presence of an underlying endogenous T-cell response to Twist in BALB/c mice. However, the increase in lytic capacity of CD8^+^ T cells taken from MVA-TWIST/TRICOM treated mice was similar for both BALB/c and C57BL/6 mice (Figure 2E and 2F), indicating that this vaccine has a similar immune-stimulatory effect regardless of mouse strain. Further evaluation of the splenic immune cell composition of BALB/c mice revealed an increase in effector memory and Twist-specific CD8^+^ T cells (Table 1) supporting the observed increase in CD8^+^ T cell functionality in the BALB/c mouse. The observed increase in activated dendritic cells in MVA-TWIST/TRICOM treated mice (Table 1) indicated a possible mechanism for this increase in highly functional Twist-specific CD8^+^ T cells. The observation that immune responses against what is considered an “undruggable” target could be induced by vaccination with MVA-TWIST/TRICOM was quite intriguing. By expressing it as a transgene from MVA, Twist was uniquely produced in the cytoplasm of infected cells leading to peptide presentation at the cell surface and, with the aid of TRICOM, the stimulation of a robust immune response.

Many studies have indicated that there is significant alteration in the functionality of the immune system in patients with cancer [33, 34, 43]. In order to determine if MVA-TWIST/TRICOM was capable of inducing Twist-specific immune responses in the setting of tumor induced immune dysfunction, immune function assays were conducted in MVA-TWIST/TRICOM treated and untreated 4T1 tumor bearing BALB/c mice. Unlike non-tumor bearing mice, 4T1-bearing mice displayed increased splenic percentages of CD4^+^ and CD8^+^ T cells, in addition to the increased percentages of effector and central CD8^+^ T cells (Tables 1 and 2). These alterations occurred in the absence of an increase in activated dendritic cells, however (Table 2). This is not completely surprising, given that MVA-TWIST/TRICOM has the capacity to convert any infected cell into a more competent antigen-presenting cell through its expression of the costimulatory molecules B7-1, ICAM-1 and LFA-3. In the case of tumor bearing mice, these increased splenic percentages, in response to MVA-TWIST/TRICOM administration, correlated with increased tumor infiltration of CD8^+^ T cells, as well as effector and central CD8^+^ T cells even in the absence of altered total lymphocytic infiltration (Figure 4A and 4B). When the functionality of splenic immune cells from tumor bearing mice was examined, it was observed that 4T1-bearing mice treated with MVA-TWIST/TRICOM exhibited improved CD4^+^ T-cell proliferation and CD8^+^ T-cell secretion of IFN-γ in response to Twist peptide compared to untreated animals (Figure 3A and 3B). The degree of CD4^+^ T cell proliferation achieved in response to Twist peptide after treatment with MVA-TWIST/TRICOM was very similar between tumor bearing and non-tumor bearing animals, however, the degree of background response was much higher in non-tumor bearing mice (Figures 2A and 3A). In addition, the degree of IFN-γ release was much lower in tumor-bearing mice compared to non-tumor bearing mice (Figures 2C and 3B). These observations support the concept of tumor-mediated immune dysfunction and display the suppressive environment in which MVA-TWIST/TRICOM is successful at inducing an immune response. The ability of MVA-TWIST/TRICOM to stimulate a successful antitumor immune response is further supported by its ability to induce antigen cascade. MVA-TWIST/TRICOM treated BALB/c mice bearing 4T1 tumors exhibited improvement in IFN-γ secretion in response to the AH-1 peptide (Figure 3C). AH-1 is the BALB/c recognized epitope of the endogenous GP70 antigen, which is expressed by the 4T1 cell line but is not included in the MVA-TWIST/TRICOM vaccine. The ability of MVA-TWIST/TRICOM to induce antigen cascade is highly significant as it has been suggested that the ability to stimulate immune responses to cascade antigens correlates better with antitumor efficacy than the ability to induce an immune response to the antigen being delivered by the vaccine [44, 45].

Previous studies have demonstrated the importance of Twist’s role in the metastatic process in the 4T1 model of metastatic breast cancer [1, 46]. Innovative studies from the same group showed that treatment of 4T1 cells with the antibiotic salinomycin, a blocker of EMT, inhibited their ability to efficiently form tumors [47]. Here we have shown in this model that vaccination with MVA-TWIST/TRICOM exhibits efficacy even when used to treat established tumors. Administration of this vaccine significantly reduced both primary 4T1 tumor growth and metastasis when administered 4 days post-tumor implantation compared to no treatment or vaccination with MVA-TRICOM (Figure 5A and 5B). When treatment was postponed to 7 days post-tumor implantation to mimic more advanced disease, primary tumor growth was still retarded (Figure 4B, insert); however, further delay of treatment resulted in no significant reduction in primary tumor growth (Figure 6C). This was not the case with metastatic disease. Regardless of the delay in treatment with MVA-TWIST/TRICOM, there was a significant reduction in the degree of lung metastasis (Figure 6E and 6F). One explanation for this observation is the significant increase in Twist expression exhibited by the lung metastases (Figure 5A, insert), which presumably made them more favorable targets for T-cell-mediated lysis. It has been observed that cells from human breast cancer metastases display increased Twist expression, suggesting that a vaccine targeting Twist would more specifically target the destruction of metastatic cells [1]. The data presented herein highlight the control of metastasis, which is a unique feature of targeting Twist. In situations where primary tumors may be removed by surgery, the ability to target metastases by the immune system is highly applicable. Since metastatic disease is extremely difficult to manage, especially in the setting of breast cancer, a vaccine targeting Twist may be of significant clinical importance. Of note, there was a significant increase in clonogenic metastatic cells in the lungs of 4T1 tumor-bearing mice when lungs were not harvested until the average group tumor volume breached 1000 mm^3^ (Figure 6D), compared to the previous experiment where all lungs were harvested on the same day when tumor volumes were significantly below 1000 mm^3^ (Figure 3B), illustrating the immense metastatic capability of this model.

Androgen deprivation therapy is a standard of care for prostate cancer [48, 49]. However, most patients eventually develop CRPC. It has been demonstrated that CRPC remains dependent on androgen signaling for growth and that CRPC is sensitive to further manipulation of androgen signaling [50]. Enzalutamide is an FDA-approved AR antagonist that blocks androgens from binding to the AR and prevents nuclear translocation and coactivator recruitment of the ligand-receptor complex. The utility of enzalutamide has been demonstrated in clinical trials [51–53], including the AFFIRM trial where it mediated a 4.8-month advantage in overall survival compared to placebo [53]. Here, focusing on mice bearing moderately differentiated to poorly differentiated adenocarcinoma, MVA-TWIST/TRICOM alone significantly improved survival in the TRAMP-Tg model of spontaneous prostate cancer. However, combining the vaccine with the AR antagonist enzalutamide not only significantly increased survival in this model, but the median overall survival was not reached before termination of the study, indicating the high degree of efficacy of this therapeutic regimen (Figure 7). This contrasts with studies of the yeast-based Twist vaccine, which did not show efficacy as a monotherapy and displayed less efficacy when combined with enzalutamide in this model, even with the administration of many more vaccinations (18 vs. 3) [35]. Enzalutamide has previously been shown to mediate thymic regeneration as well as an increase in the sensitivity of TRAMP C2 cells to immune-mediated killing [35]. This immunomodulatory capacity of enzalutamide partially explains the data reported here and supports the clinical evaluation of ADT in combination with active immunotherapy for the treatment of metastatic prostate cancer.

Although the data presented here demonstrate the successful targeting of Twist for the treatment of cancer, it is unclear whether Twist is a valid target for immunotherapy or other types of cancer treatment. Twist expression has been identified in several normal human adult tissues including the testis, ovary, placenta, thymus, colon, lung and brain, possibly reducing its ability to be targeted without detriment to the patient [21]. The transcription factor brachyury has been shown to play a major role in EMT, a key process in the dissemination of cancer cells, leading to the formation of distant metastases, in a wide range of human carcinomas [4, 31, 54, 55]. In addition, brachyury is differentially expressed in human carcinomas vs. normal human adult tissue, perhaps making it a safer target than Twist in humans [21]. However, the lack of brachyury expression in the mouse makes it difficult to evaluate the efficacy of vaccines targeting this transcription factor [21]. The functional homology between brachyury and Twist, however, allows vaccines targeting Twist to serve as models for vaccines targeting brachyury in preclinical murine tumor models. Based on the preclinical data obtained by using Twist as a model target antigen, two brachyury vaccines are currently being evaluated clinically, including an MVA-TRICOM-based brachyury vaccine (MVA-brachyury/TRICOM) expressing human transgenes for B7-1, ICAM-1 and LFA-3 [56, 57].

## MATERIALS AND METHODS

### Animals

Eight- to 12-week-old female BALB/c and C57BL/6 mice were obtained from the National Cancer Institute’s Frederick Cancer Research Facility, Frederick, MD. TRAMP-Tg mice on the C57BL/6 background were bred and maintained at the National Institutes of Health (Bethesda, MD) [58]. Age-matched male TRAMP-Tg mice were used for the antitumor study. Mice were housed and maintained in microisolator cages under specific pathogen-free conditions in accordance with Association for Assessment and Accreditation of Laboratory Animal Care guidelines. All experimental studies were approved by the National Cancer Institute’s Intramural Animal Care and Use Committee.

### Tumor cells

4T1 murine metastatic breast carcinoma, MC38 murine colon carcinoma and P815 murine lymphoblast-like mastocytoma cell lines were purchased from American Type Culture Collection (Manassas, VA) and cultured in complete medium (RPMI or DMEM supplemented with 10% fetal bovine serum, 2 mM glutamine, 1 mM HEPES buffer, 50 μg/mL gentamicin, 100 IU/mL penicillin, 100 μg/mL streptomycin and 300 μg/mL G418) at 37°C/5% CO_2_.

### Poxviral vaccine constructs

MVA containing transgenes for the murine costimulatory molecules B7-1, ICAM-1 and LFA-3 (MVA-TRICOM) has been previously described [22]. MVA-TWIST/TRICOM was designed, constructed and manufactured by Bavarian Nordic (BN) under a Cooperative Research and Development Agreement with the National Cancer Institute. Twist was optimized for expression and MVA-BN was used as the backbone [59]. For *in vivo* studies, two to three weekly doses of 1 × 10^8^ infectious units/mouse/dose of MVA-TWIST/TRICOM were administered s.c [24].

### Enzalutamide administration

Enzalutamide (Selleck Chemicals, Houston, TX) was admixed with standard rodent diet (Research Diets Inc., New Brunswick, NJ) at a concentration of 85.7 mg/kg of diet in order to deliver 10 mg/kg bw/day to the animals, a dose that achieves clinical serum concentrations [35]. Animals began receiving enzalutamide diet on day 0 and continued on the diet for the duration of the study.

### Western blotting

Twist expression was determined by western blot using rabbit polyclonal antibodies to Twist (Abcam, Cambridge, MA) and GAPDH (Cell Signaling, Danvers, MA). MC38 cells treated with 10 MOI (multiplicity of infection) MVA-TRICOM or MVA-TWIST/TRICOM, or left untreated for 24 hours were lysed using Cell Lysis Buffer containing 1 mM PMSF (Cell Signaling) and 10 μL/mL HALT Protease/Phosphatase Inhibitor Cocktail (Thermo Scientific, Rockford, IL) according to the manufacturer’s protocol. Protein concentration was measured using a BCA Protein Assay Kit (Thermo Scientific). Aliquots containing 50 μg of protein were run on a Bolt 4%–12% gradient Bis-Tris gel in a Bolt Mini Gel Tank and transferred to a PVDF membrane using the iBLOT Transfer System (Life Technologies, Grand Island, NY). Membranes were blocked for 1 hour at room temperature with PBS containing 5% BSA and 0.05% Tween20, then incubated with primary antibodies overnight at 4°C in block. Membranes were then incubated with an IRDye-labeled goat anti-rabbit secondary antibody (LI-COR Biosciences, Lincoln, NE) at a 1:10000 dilution in block for 1 hour at room temperature. Membranes were imaged using the Odyssey Infrared Imaging System (LI-COR Biosciences).

### Flow cytometry

Expression of B7-1, ICAM-1, and LFA-3 was determined by flow cytometry. Treated MC38 cells were stained with FITC-labeled antibodies to CD80 (B7-1), CD54 (ICAM-1) and CD48 (LFA-3) (BD Biosciences, San Jose, CA). Cells were incubated with antibodies for 30 min at 4°C. Samples were acquired on a FACScan flow cytometer (Becton Dickinson, Franklin Lakes, NJ). The effect of MVA-TWIST/TRICOM on splenic immune cell populations was examined in tumor bearing and non-tumor bearing BALB/c mice 17 days after receiving two vaccinations with MVA-TWIST/TRICOM or being left untreated (*n* = 5/group). Vaccination of tumor bearing mice began 4 days post-implantation of 5 × 10^4^ 4T1 mammary tumor cells. Spleens were prepared and stained as described previously [60], using the following antibodies: CD3e-V500, t-APC, CD8a-Pacific Blue, CD25-FITC, CD44- PerCP-Cy5.5 CD11b-V500, Gr-1-APC, CD11c-PerCP-Cy5.5, CD40-FITC (BD Biosciences); CD62L-PE-Cy7, FoxP3-PE, MHC II-efluor450 (eBioscience, San Diego, CA); and CD49b-PE-Cy7 (Biolegend, San Diego, CA). Tetramer staining (Beckman Coulter, Pasadena, CA) was performed on splenocytes from non-tumor bearing mice following 7 days of *in vitro* stimulation with 1.0 μg/mL Twist peptide (BALB/c - LYQVLQSDEL). All samples were acquired on a BD Verse flow cytometer. All marker expression was determined using FlowJo software (TreeStar, Inc., Ashland, OR).

### Murine CD4^+^ T-cell proliferation

To assess the effect of MVA-TWIST/TRICOM on CD4^+^ T-cell proliferation, in non-tumor bearing mice, female BALB/c or C57BL/6 mice (*n* = 5/group) were given three weekly vaccinations or left untreated. Three weeks following the last vaccination, mice were euthanized and splenocytes were collected and pooled. For analysis in tumor bearing mice, BALB/c mice were administered two weekly vaccinations or left untreated beginning 4 days after implantation of 5 × 10^4^ 4T1 mammary tumor cells. Splenocytes were collected and pooled 17 days following the last vaccination. CD4^+^ T cells were purified and Twist-specific proliferation was measured as described previously [32]. Briefly, the level of CD4^+^ T-cell proliferation was determined by [^3^H]-thymidine incorporation in the presence of irradiated APCs (2000 rad) and Twist peptide (BALB/c - QQPASGKRGARKRRS, 6.25 μg/mL; 4T1 tumor-bearing BALB/c - 12.5 μg/mL; C57BL6 - FSVWRMEGAWSMSAS, 5.0 μg/mL). Cells were harvested using a Tomtec cell harvester (Wallac Inc., Gaithersburg, MD). [^3^H]-thymidine incorporation was measured using a Wallac 1205 Betaplate MicroBeta counter (Wallac Inc.).

### Murine CD8^+^ T-cell responses

To assess the effect of MVA-TWIST/TRICOM on CD8^+^ T-cell activity, in non-tumor bearing mice, female BALB/c or C57BL/6 mice (*n* = 5/group) were given three weekly vaccinations of MVA-TRICOM or MVA-TWIST/TRICOM, or left untreated. Three weeks following the last vaccination, mice were euthanized and splenocytes were collected and pooled. For analysis in tumor bearing mice, BALB/c mice were administered two weekly vaccinations or left untreated beginning 4 days after implantation of 5 × 10^4^ 4T1 mammary tumor cells. Splenocytes were collected and pooled 17 days following the last vaccination. The level of CD8^+^ T-cell activity was determined by the amount of secreted IFN-γ following 7 days of *in vitro* stimulation with 1.0 μg/mL Twist peptide (BALB/c - LYQVLQSDEL [61], C57BL6 - TQSLNEAFL [35]). The response to a non-vaccine encoded self-antigen, GP70, was also analyzed in tumor bearing BALB/c mice using the AH-1 peptide (SPSYVYHQF). Interferon (IFN)-γ production from pooled splenocytes was measured as previously described [32]. Data were acquired using a FACScan flow cytometer and analyzed using BD CBA analysis software (Becton Dickinson). T-cell lytic capacity was also measured as previously described [32]. Briefly, following 6 days of *in vitro* stimulation with 1.0 μg/mL Twist peptide, splenocytes from non-tumor bearing mice were incubated for 5 hours with indium-111 labeled p815 target cells pulsed with 12.5 μg/mL Twist peptide. Lytic capacity was calculated as [(experimental cpm – spontaneous cpm) / (maximum cpm – spontaneous cpm)] × 100.

### Tumor immunohistochemistry and flow cytometry

BALB/c mice (*n* = 5/group) were administered two weekly vaccinations or left untreated beginning 4 days following 4T1 tumor implantation. On day 28, post-tumor implantation, tumors were harvested with a portion of each tumor being fixed with Z-Fix (Anatech Ltd., Battle Creek, MI) and the rest being dissociated according to the protocol for preparation of single-cell suspensions from implanted mouse tumors (Miltenyi Biotec San Diego, CA). Fixed tumor sections were stained with a rabbit polyclonal antibody to CD3 (Dako) at 1:1000. Control sections were stained with a matched isotype antibody. Entire tumor sections were digitally scanned by an Aperio ScanScope CS scanning system and analyzed by Aperio ImageScope Viewer software (Aperio Technologies Inc., Vista, CA). Positive cells were identified using the Positive Pixel Count v9 algorithm. Dissociated tumor was stained for flow cytometric analysis using the same antibodies and protocol as in the splenic immune cell population analysis.

### RNA isolation, quantitative RT-PCR

4T1 primary tumors and lung metastases were isolated from untreated BALB/c mice (*n* = 3) and prostates were collected from untreated TRAMP-Tg and wild-type C57BL/6 mice (*n* = 3 from each age group). Cell lysates were obtained, RNA was isolated and quantitative RT-PCR was performed as previously described [32]. PCR was performed on the 7300 Real-Time PCR System (Applied Biosystems, Waltham, MA). Twist expression is represented relative to GAPDH, the endogenous control.

### Antitumor studies

For 4T1 orthotopic tumor studies, female BALB/c (*n* = 10–15/group) were injected s.c. with 5 × 10^4^ 4T1 mammary tumor cells. Four, 7 or 15 days post-tumor implantation, mice received 2–3 weekly injections of MVA-TRICOM or MVA-TWIST/TRICOM, or were left untreated. Primary tumor dimensions were measured twice weekly and tumor volumes were calculated as (length × width^2^)/2. Mice were euthanized either on day 21 or when the average tumor volume in each group breached 1000 mm^3^. The degree of lung metastasis was determined as described previously [32]. Briefly, lungs were homogenized to a single cell suspension using gentleMACS™ C Tubes (Miltenyi Biotec); cells were then plated in the presence of 6-thioguanine. After 10–14 days, colonies were enumerated yielding “clonogenic metastatic cells/lung.” For TRAMP-Tg studies, male TRAMP-Tg mice were divided into four age-matched groups (*n* = 13–16/group, aged 20–36 weeks; representing moderately differentiated to poorly differentiated adenocarcinoma [35]) and received (a) no treatment, (b) MVA-TWIST/TRICOM alone, (c) enzalutamide alone and (d) MVA-TWIST/TRICOM plus enzalutamide. Mice treated with MVA-TWIST/TRICOM received three weekly vaccinations; mice treated with enzalutamide were fed enzalutamide-containing diet throughout the study.

### Statistical analysis

GraphPad Prism 5 statistical software (Version 5; GraphPad Software, La Jolla, CA) was used to measure 2-tailed unpaired Student’s *t* tests for differences between groups, with a 95% confidence interval. All data with error bars represent the mean ± S.E.M. for the indicated number of replicates or individual mice. FlowJo software was used to determine significant differences in the distribution of flow cytometry data using the Kolmogorov-Smirnov test.
